# Body composition is related to cognitive function among young adults in Ghana

**DOI:** 10.1186/s40359-024-01569-0

**Published:** 2024-03-01

**Authors:** Linda Fabea, Freda Dzifa Intiful, Irene E. Hatsu, Joana Larry-Afutu, Laurene Boateng

**Affiliations:** 1 Department of Dietetics, University of Ghana, Accra, Ghana; 2 Department of Human Sciences; College of Education and Human Ecology, Ohio State University, Columbus, OH USA; 3 Department of Psychology, University of Ghana, Accra, Ghana

**Keywords:** Cognitive function, Obesity, Trail making test, Adiposity

## Abstract

**Background:**

A growing body of evidence suggests that obesity can affect cognitive function. However, it is unclear whether this effect is independent of obesity-related comorbidities. This study thus sought to determine the association between body composition and cognitive function of young adults in Ghana with less predisposition to obesity-related comorbidities.

**Methods:**

A cross-sectional study design was employed, involving 381 participants recruited by simple random sampling. After consenting, participants completed questionnaires that assessed sociodemographic characteristics, along with assessments for anthropometric measures and cognitive function. Analyses for associations were conducted by either Pearson’s correlation test or chi-Square test of independence.

**Results:**

Over half (60%) of participants were females and 69.6% were in the first year with a mean age of 20.18 ± 2.52 years. Based on Pearson’s correlation test, no significant association was found between Body Mass Index (BMI) and Waist to Hip Ratio (WHR), and Trail Making Test-A and B (TMT-A and TMT-B). Nonetheless, a chi-square test showed a significant association, between BMI and TMT-A (*p* = 0.01), and WHR and both TMT-A (*p* = 0.001) and TMT-B (*p* = 0.02). Weak direct correlations were found between body fat percentage and TMT-A (*r* = 0.120, *p* = 0.019) and TMT-B (*r* = 0.133, *p* = 0.009). Further, a weak inverse correlation was found between muscle mass and TMT-A (*r* = − 0.141, *p* = 0.006) and TMT-B (*r* = − 0.144, *p* = 0.005).

**Conclusion:**

High body fat, low muscle mass and body fat distribution may have a significant association with cognitive functions and must be considered in obesity interventions. This study provided more insight on the association between BMI and cognitive function and would be helpful in designing new weight management interventions or modifying existing interventions to consider the influence of obesity on cognitive function.

## Background

Obesity and overweight are medical conditions characterised by the accumulation of excessive fat in the body [1]. They can be assessed using anthropometric measurements and indices such as body mass index (BMI), body fat percentage and waist to hip ratio (WHR) [2]. However, BMI is the most widely used anthropometric index due to its simplicity [2]. Studies have however shown that BMI alone is not enough and should not be the only parameter used in diagnosing obesity [3]. This is due to reports indicating that individuals with a normal BMI can still maintain a high body fat percentage, a scenario termed ‘normal weight obesity’. This situation may elevate the risk of metabolic disorders. Body fat and muscle mass percentage can be determined using Bioelectric Impedance Analyser (BIA), Dual-energy X-ray Absorptiometry (DXA) etc. [3].

The increasing prevalence of overweight and obesity worldwide, has become an issue of public health concern. Ghana is no exception as recent studies show that 43% of Ghanaian adults are overweight (25.4%) or obese (17.1%) [4]. Research shows that higher BMI is not only associated with an increased risk of comorbidities such as cardiovascular disease, diabetes mellitus, musculoskeletal and respiratory problems, but also with cognitive function deficits [1, 5]. Cognitive function cuts across perception, attention, memory and executive function and can be evaluated using psychological assessment tests such as the Wisconsin sorting task and Trail Making Test (TMT) among others [6]. Research has further demonstrated that, obesity usually affects brain structure and function, particularly working memory and executive function [6–10]. Specifically, obesity has been linked to structural brain abnormalities, a decrease in performance on executive function tests, as well as hippocampal and frontal lobe dysfunction [6–10]. Moreover, elevated BMI is associated with a decreased brain volume independent of age and morbidity, grey and white matter atrophy in the frontal, temporal and occipital cortices as well as the hippocampus, thalamus and midbrain [11]. Other longitudinal studies have shown that overweight and obesity in adulthood is associated with increased risks of cognitive dysfunction and dementia in later life [1, 5]. Obesity is thus considered a significant risk factor for diminished neural integrity [8]. Observations made through animal and clinical studies suggest that the main mechanisms through which obesity influences cognitive functions are through impaired regulation of insulin, glucose and leptin, increased systemic and central inflammation, increased brain atrophy, metabolic dysfunction and cardiovascular diseases [5].

The above notwithstanding, other studies have proposed a bidirectional relationship between obesity and cognitive function [6]. Excess body fat, especially abdominal fat, can lead to inflammation and metabolic changes that may impair brain function [6]. On the other hand, cognitive function can also influence obesity. For example, individuals with poor impulse control, lower executive function, or emotional issues may have difficulties with self-control and may be more likely to engage in unhealthy eating habits, leading to obesity. Additionally, cognitive function can impact an individual’s ability to engage in physical activity, which is crucial for maintaining a healthy weight. Thus, obesity could in part be a neurological condition [6].

It is worth noting that the aforementioned studies as well as existing literature focus mostly on older or middle-aged adults who may be experiencing other comorbidities associated with high BMI such as diabetes and cardiovascular disease. Although some studies have suggested that cardiovascular disease may lead to cognitive deficits [12], other systematic reviews have also questioned whether the association between obesity and cognitive impairment is independent of obesity-related comorbidities [12]. In light of the lower prevalence of obesity-related comorbidities among young adults below 30 years compared to older adults, it is important to ascertain whether or not the impact of obesity on cognitive function is evident among young adults [5]. Thus, Cook and colleagues suggested that to study the association between high BMI and cognitive function, it may be advisable to recruit young adults who have no apparent metabolic abnormalities and cardiovascular diseases in order to reduce the confounding factors in the study [12].

Interestingly, this body of evidence is mostly reported in developed countries with little or no studies done in Africa and Ghana specifically. With the increasing prevalence of obesity in Ghana, it is important to examine the relationship between obesity and cognitive function to prevent double burden of disease. To the best of our knowledge, this is the first study to examine the association between obesity and cognitive function in Ghana and to also recruit young adults with no reported comorbidities of obesity. Findings of the study would be helpful in designing new weight management interventions or modifying existing interventions which would consider the influence of obesity on cognitive function. Further, the study would add to the existing body of literature on this subject.

This study thus aimed to examine the relationship between body composition and cognitive function among young adults in Ghana, with less predisposing factors to cognitive decline and obesity-related comorbidities. We hypothesize that, regardless of the presence of obesity-related comorbidities, obesity and adiposity negatively affect the cognitive function of individuals. Other potential confounding factors such as age and educational level were addressed in the careful selection of study participants.

## Methods

### Aim, design, and setting of study

The aim of the study was to examine the relationship between body composition and cognitive function among young adults in Ghana, with less predisposition to obesity-related comorbidities. A cross-sectional study was designed and was conducted on the main campus of the University of Ghana (UG) between March to May 2021. UG is located 13 km northeast of Accra, with a population of about 53,7643 [13]. The University has a total of 15 halls of residence classified into three groups: traditional halls, University of Ghana Enterprises Limited (UGEL) halls and UG private hostels based on their geographical location. For this study, eight mixed halls were selected. All UG undergraduate students aged 18 to 30 years, and willing to provide written informed consent were eligible to participate in this study. Individuals who had been diagnosed with any mental or neurological condition especially dementia, and those with any disability that would affect anthropometric measurements were excluded from the study. This was done based on self-reported medical history.

### Measures

A modified WHO stepwise semi-structured questionnaire was used to collect data on demographic characteristics and TMT was used to collect information on cognitive functions of participants. Data was collected through face-to-face interview with participants. As data collection was carried out during the COVID-19 era, all COVID-19 protocols were observed to ensure participants and research staff safety. The following measures were put in place to prevent the spread of COVID-19 during and after data collection:

- It was ensured that all individuals present at the data collection site were wearing nose masks.
- It was also ensured that a minimum of 1 metre social distance was observed among participants and research team members.
- All data collection tools were sanitised with 70% ethanol before and after each use.
- It was ensured that all participants and research team members sanitised their hands with 70% ethanol before and after handling any data collection tool.

#### Anthropometric and body composition assessment

Participants height were measured using a stadiometer (Seca 213). To do this, each participant was asked to stand barefooted and straight against the backboard of the stadiometer, heels together and toes apart. Without any hair ornaments participant’s head was aligned in a Frankfort horizontal plane as they looked straight ahead. With the stadiometer headpiece lowered gently to firmly rest on the top of participant’s head, height measurement was recorded to the nearest 0.1 cm. Body weight and composition (percentage body fat and total muscle mass) were measured using Omron BF511. This equipment measures body composition via the Bioelectrical impedance analysis (BIA) method. BIA measures body composition based on the rate at which a low voltage electrical current travels through the body. Body fat (adipose tissue) causes greater resistance (impedance) than lean mass and slows the rate at which the current travels. A built-in software then estimates the various body components in the appropriate units. The BIA measurement was done with participants wearing single layered clothing and without shoes. After stepping on the scale, measurement was done with each participant holding the monitor at an angle of 90°. Weight was measured to the nearest 0.01 kg; body fat percentage and muscle mass percentage were further categorised based on the cut-offs for this age group (18–30 years) provided in the manual [14, 15]. BMI was calculated based on standard formula (BMI = weight(kg)/height(m)^2^) and categorised based on WHO cut-offs [16]. To measure waist and hip circumference, an adjustable measuring tape was extended around the waist (at the umbilicus) for waist circumference and the widest part of the hips respectively for hip circumference. Measurements were recorded to the nearest 0.1 cm. All measurements were taken in duplicate and the average calculated to ensure accuracy. Waist -to-Hip ratio (WHR) was calculated and categorised by sex into normal or obese based on WHO cut-offs [16].

#### Cognitive function assessment

The trail making test (TMT) was used to determine the cognitive function of study participants [17]. The TMT has 2 parts (A and B), both of which consist of 25 circles. In part A, participants were asked to draw lines to connect the circles with numbers from 1 to 25, as quickly as possible without lifting the pen or pencil from the paper. Errors were immediately pointed out for correction as needed. This activity was timed and recorded. The same procedure was repeated for part B, but with this, the participant alternated between numbers (1–13) and alphabets (A –L). A score of ≤29 seconds was considered adequate performance while ≥78 seconds was considered an impaired cognitive function on TMT-A. Likewise, on TMT-B, a score of ≤75 seconds indicated adequate performance with a score ≥ 273 seconds indicating significant impairment in cognitive function [17].

### Sample determination and statistical analyses

Sample size was calculated using the Cochran sample size calculation formula for finite population [18]. This assumed that 50% of the study population would exhibit the characteristic of interest and allowed a greater level of variability. Using a 95% confidence interval, a 5% error margin and a 5% attrition rate, a sample size of 381 was yielded.

Simple random sampling technique was used to select study participants. All 15 halls present on the university campus were assigned numbers except the graduate students’ hostel. Eight halls were selected using simple random sampling (lottery method). Using the same method, all rooms were assigned numbers, and 25 students were selected from each hall. Likewise, two students were selected at random from each room previously picked.

Data management and analyses were performed using Statistical Package for Social Sciences (SPSS) version 20. Descriptive statistics were used to categorise socio-demographic characteristics and were presented as frequencies and percentages while continuous variables such as anthropometric data, cognitive function data etc. were presented as means and standard deviations. Pearson’s correlation test and Chi-square test of independence were used to determine the association between anthropometric indices, and cognitive function of participants.

### Ethics approval and consent to participate

Ethical approval was obtained from the University of Ghana College of Health Science Ethics and Protocol Review Committee (CHS-Et/M.5–4.9/2020–2021). Informed written consent was also sought from participants. All methods were performed in accordance with the relevant guidelines and regulations of the University of Ghana College of Health Science Ethics and Protocol Review Committee**.** Data was kept confidential and access to data was limited to only the research team. In addition, participants were identified by codes instead of names.

## Results

### Sociodemographic characteristics

Table 1 shows the sociodemographic characteristics of participants. Majority (60%) of the participants were females. A total of 381 participants were included in this study. The mean age was 20 ± 2.52 years and males were older than females (*p* = 0.001) Most participants were of Ghanaian nationality (99%) and were in the first year of their university education. A large proportion (91%) of the participants lived with their nuclear families with a mean family size of 6.

**Table 1:** Sociodemographic characteristics of participants

Variable	Total Mean ± SD	Male Mean ± SD	Female Mean ± SD
Age (years)	20.18 ± 2.52	20.70 ± 3.24	19.84 ± 1.84
Family size	6.31 ± 3.09	6.09 ± 2.32	6.47 ± 3.51
	Total N(%)	Male n(%)	Female n(%)
Sex	381 (100)	152 (39.9)	229 (60.1)
**Hall of residence:**			
Hall I	49(12.9)	36(23.7)	13(5.7)
Hall II	35(9.2)	16(10.5)	19(8.3)
Hall III	90(23.6)	31(20.4)	59(25.8)
Hall IV	32(8.4)	11(7.2)	21(9.2)
Hall V	34(8.9)	12(7.9)	22(10.6)
Hall VI	32(8.4)	7(4.6)	25(10.9)
Hall VII	44(11.5)	17(11.2)	27(11.8)
Hall VIII	65(17.1)	22(14.5)	43(18.8)
**Nationality:**			
Ghanaian	377(99.0)	151(99.3)	226(98.7)
Non-Ghanaian	4(1.0)	1(0.7)	3(1.3)

*SD* Standard deviation

### Participant anthropometric and body composition indices

As shown in Table 2, males had a higher mean weight, height, muscle mass percentage, waist circumference and WHR than the females (All *p* < 0.01). However, females had greater body fat percentage than males (*p* < 0.001). BMI and hip circumference were not significantly different between the two groups.

**Table 2:** Anthropometric indices of participants

Variable	Total Mean ± SD	Male Mean ± SD	Female Mean ± SD	*P*-value
Weight (kg)	64.80 ± 12.16	66.89 ± 12.43	63.41 ± 11.80	0.006*
Height (m)	1.64 ± 0.07	1.68 ± 0.08	1.62 ± 0.06	< 0.001*
BMI (kg/m^2^)	24.06 ± 3.84	23.73 ± 3.61	24.27 ± 3.98	0.178
Body fat (%)	29.93 ± 9.81	25.05 ± 10.67	33.17 ± 7.64	< 0.001*
Muscle mass (%)	31.67 ± 6.66	34.95 ± 8.11	29.50 ± 4.30	< 0.001*
Waist circumference (cm)	76.91 ± 8.82	78.43 ± 8.50	75.92 ± 8.91	0.006*
Hip circumference (cm)	98.43 ± 1–.70	97.76 ± 10.35	98.88 ± 10.91	0.320
WHR	0.78 ± 0.07	0.81 ± 0.08	0.77 ± 0.07	< 0.001*

*P-value < 0.05, 95% confidence interval, *WHR* waist-to-hip ratio

Table 3 shows the anthropometric and body composition indices of study participants. More than half of the participants (67.5%) had a normal BMI and less than a quarter were overweight. An equal proportion of males and females were underweight, however, females were overrepresented in the obese category (7.9%), while more males were overweight (27.0%). More than half of the participants had high body fat percentage, especially among males (66.4%), compared to females (48.5%). In addition, over 50% of females had a normal muscle mass percentage compared to only 16% of males; almost half of the males had a low muscle mass percentage (48%). Based on WHR, however, 14% of females compared to about 5% of males were at an elevated risk of cardiometabolic comorbidities.

**Table 3:** Comparison of body composition indices by sex

Variable	Total *N* (%)	Male *n* (%)	Female *n* (%)
**BMI**			
Underweight	10(2.6)	5(3.3)	5(2.2)
Normal	257(67.5)	100(65.8)	157(68.6)
Overweight	90(23.6)	41(27.0)	49(21.4)
Obesity	24(6.3)	6(3.9)	18(7.9)
**Body fat percentage**			
Low	19(5.0)	8(5.3)	11(4.8)
Normal	39(39.4)	43(28.3)	107(46.7)
High	212(55.6)	101(66.4)	11(48.5)
**Muscle mass percentage**			
Low	94(24.7)	73(48.0)	21(9.2)
Normal	144(37.8)	24(15.8)	120(52.4)
High	143(37.5)	55(36.2)	88(38.4)
**WHR**			
Normal	341(89.5)	145(95.4)	196(85.6)
Elevated	40(10.5)	7(4.6)	33(14.4)

### Cognitive function of participants

The average processing speed based on TMT-A scoring system is 29 seconds [17]. In this study, only 20% of the population scored less than 29 seconds, comprising 23.8% of males and 18.3% of females (Table 4). Majority (65.4%) of the participants scored above 29 seconds which depicts a reduced processing speed (classified as a score of ≥78 seconds) but not an impairment in cognitive function. About 14% of the population were classified as impaired (17.9% of females vs. 8.6% of males). For TMT-B, nearly 43% of participants scored below the required average score of 75 seconds, indicative of high cognitive function (51.3% of males vs. 37.1% of females). Only 2.1% of the population were classified as impaired (3.1% of females vs. 0.7% of males) (Table 4).

**Table 4:** Participants’ cognitive function based on the TMT

Variable	Total *N* (%)	Male *n* (%)	Female *n* (%)	*P*-value
**TMT-A (seconds)**				
Mean ± SD	51.02 ± 34.28	44.62 ± 23.78	55.27 ± 39.23	0.003*
High processing speed (< 29 secs)	78(20.5)	36(23.7)	42(18.3)	
Average (29 secs)	0(0)		0(0)	
Low processing speed (< 78 secs but > 29 secs	249(65.4)	0(0) 103(67.8)	146(63.8)	
Impaired processing speed (> 78 secs)	54(14.2)	13(8.6)	41(17.9)	
**TMT-B (seconds):**				
Mean ± SD	99.86 ± 69.23	87.37 ± 48.38	108.15 ± 79.14	0.004*
High executive function (<75secs)	163(42.8)	78(51.3)	85(37.1)	
Average (75secs)	0(0)	0(0)	0(0)	
Low executive function (<273secs but >75secs	210(55.1)	73(48.0)	137(59.8)	
Impaired executive function (>273secs)	8(2.1)	1(0.7)	7(3.1)	

*P-value < 0.05; 95% confidence interval

### Association between cognitive function and anthropometric indices of participants

Based on results from the Pearson’s correlation test, no significant associations were found between BMI and both TMT-A and TMT-B; this finding was similar for WHR (Table 5). A weak direct correlation was, however, found between body fat percentage and both TMT-A and TMT-B. Further, a weak inverse correlation was found between muscle mass and TMT-A and TMT-B (Table 5).

**Table 5:** Association between cognitive function and anthropometric indices of participants

Variables	*R* value; *P* value	
	TMT-A	TMT-B
Body fat percentage	*r* = 0.120, *p* = 0.019	*r* = 0.133 *p* = 0.009
Muscle mass percentage	***r*** **= -0.141,** ***p*** **= 0.006**	***r*** **= -0.144,** ***p*** **= 0.005**
BMI	*r* = −0.070, *p* = 0.170	*r* = −0.057, *p* = 0.263
WHR	*r* = −0.012, *p* = 0.809	r = 0.060, *p* = 0.240

*P value< 0.05

$$\textbf{r}=+/-<\textbf{0.39}=\textbf{weak},\kern0.75em \textbf{r}=+/-\textbf{0.4}-\textbf{0.59}=\textbf{moderate},\kern1em \textbf{r}=+/-\textbf{0.6}-\textbf{0.79}=\textbf{strong},\kern1.25em \textbf{r}=+/-\textbf{0.8}-\textbf{1}=\textbf{very}\ \textbf{strong}$$

Table 6 shows the chi square test of association of TMT-A, with BMI and WHR. Statistically significant non-linear associations were found between BMI and TMT-A, and between WHR and TMT-A. It was found that 14.8% of the participants with normal BMI had impaired cognitive function, with 13.3% in the overweight group, 12.5% in the obese group and 10% in the underweight group all having impaired cognitive function. With regards to central obesity, 35% of participants with elevated WHR had impaired cognitive function compared to 11.7% in the normal WHR group. This gives an indication that individuals with central obesity may be more likely to have cognitive impairment (processing speed) than their non-obese (based on central adiposity) counterparts.

**Table 6:** Association between TMT-A, BMI AND WHR of participants

Variable	TMT-A			
	High cognitive function *n* (%)	Low cognitive function *n* (%)	Impaired cognitive function *n* (%)	*P*-value
**BMI**				
Underweight	4(40.0)	5(50.0)	1(10.0)	0.010*
Normal	38(14.8)	181(70.4)	38(14.8)	
Overweight	27(30.0)	51(56.7)	12(13.3)	
Obese	9(37.5)	12(50.0)	3(12.5)	
**WHR**				0.001*
Normal	72(21.1)	229(67.2)	40(11.7)	
Elevated	6(15.0)	20(50.0)	14(35.0)	

*P value < 0.05

With regards to the association of TMT-B with BMI and WHR, a statistically significant non-linear association was found between WHR and TMT-B but not BMI. Only 25% of participants with elevated WHR had high cognitive function compared to the 44.9% found in the normal group. This shows that an individual with central obesity may be more likely to have cognitive impairment (executive function) than a non-obese individual (Table 7).

**Table 7:** Association between TMT-B, BMI and WHR of participants

Variable	TMT-B			
	High cognitive function *n* (%)	Low cognitive function *n* (%)	Impaired cognitive function *n* (%)	*P*-value
**BMI**				
Underweight	7(70.0)	3(30.0)	0(0)	0.115
Normal	99(38.5)	150(58.4)	8(3.1)	
Overweight	45(50.0)	45(50.0)	0(0)	
Obese	12(50.0)	12(50.0)	0(0)	
**WHR**				
Normal	153(44.9)	181(53.1)	7(2.1)	0.02*
Elevated	10(25.0)	29(72.5)	1(2.5)	

*P value < 0.05

## Discussion

Findings of this study suggest that cognitive function, specifically, processing speed and executive function of young adults may be related to obesity indicators particularly central obesity. The prevalence of overweight and obesity found in this study is slightly lower, but consistent with a systematic review of studies in Ghana, which found that 43% of Ghanaians were overweight or obese [4]. Another cross-sectional study among University students in Ghana with an age range of 16 to 20 years reported a 23% prevalence of overweight and obesity, with higher prevalence among females than males (*p* < 0.05) [19]. Similar trends have been reported in other Africa-based studies [20–23]. In the current study, we found obesity to be higher in females than in males (*p* < 0.05). This finding is consistent with another Ghanaian cross-sectional study among 1552 university students where the prevalence of obesity among males and females were found to be 3.9 and 7.6% respectively (*p* < 0.05) [24].

Due to its limitation of inability to measure localized adiposity, BMI is not recommended as a sole obesity diagnostic tool in research [3]. In this study, Bioimpedance analysis (BIA) was used to assess percent body fat and muscle mass. Mean body fat percentage was found to be 29.93 ± 9.81%, with females having a significantly higher body fat percentage than males, in this study (*p* < 0.05). A similar pattern was observed in a study among university students in Ghana [24], and also among first year Chinese university students [25]. In this study, we recorded a high percent body fat in 55% of participants (higher in males). This is similar to findings of a study among Ghanaian women which reported a high body fat prevalence of 57% [2]. Contrary to the higher prevalence of obesity among females based on BMI, more males were obese compared to females based on percent body fat. This may be attributed to the differences in cut-offs for males and females.

Percent muscle mass comprises the weight of both skeletal muscle and smooth muscle of an individual [26]. It is a determinant of obesity and has been linked to many obesity-related comorbidities, poor psychological health, low quality of life and high risk of mortality [26, 27]. Findings from this study showed a mean muscle mass percentage of 31.67 ± 6.66%, which is similar to findings from a study in Australia among 168 participants between the ages of 18 to 35 years which recorded a mean muscle mass percentage of 30.4% [28]. As expected, males had statistically significant (*p* < 0.05). higher muscle mass than females, which is also consistent with the finding from the aforementioned Australian study [28]. Despite the males having a higher mean muscle mass compared to females, majority of males were classified as having low muscle mass (48.0%) compared to females (9.2%). More females were within the normal muscle mass category (52.4%) when compared to males (15.8%).

Unlike BMI which has the same cut-offs for both males and females in this age group, body composition analysis has different cut-off for males and females in this population. Males have a lower cut-off point for body fat percentage and a higher one for muscle mass percentage due to the differences in the anatomy and physiology of the two sexes [15, 16]. Hence, there is a possibility of high prevalence of normal weight obesity in males based on body composition analysis which could not be determined by BMI in this study. In addition, the males were significantly taller than the females which could also affect the prevalence of obesity based on BMI. This may account for the differences in the prevalences of obesity based on body composition assessment between males and females, compared to BMI [15, 16].

Central (abdominal) obesity, described as the accumulation of body fat at the trunk or around the abdominal region, was also assessed in this study using WHR. Central obesity has been identified as a major issue of public health concern and has been linked to many obesity-related comorbidities especially cardiovascular diseases etc. [29]. In addition, WHR has been observed to be a strong determinant of myocardial infarction from the INTERHEART study [30]. WHR is able to determine distribution of fat in the body and it is correlated to body fat percentage. Findings from this study reported a mean waist circumference of 76.91 ± 8.82 cm which is in line with the findings from other studies conducted among university students in Ghana and elsewhere [24, 31, 32]. Based on sex, males had a statistically significant higher waist circumference than females A similar trend was observed in the aforementioned studies done in Ghana and other countries as well [2, 24, 31–34].

For WHR, this study recorded a mean of 0.78 ± 0.07 which was lower than reported in some prior studies [2, 34]. Similar to the waist circumference, males recorded higher waist to hip ratio than females (0.81 vs. 0.77). Similar trends were noted in previous studies [2, 31, 34]. We found 10.5% of our study participants had an elevated WHR (higher proportion of females compared to males), which is lower than the findings reported in previous studies. A study from Macedonia among individuals ages 18 to 20 years reported a 45.23% prevalence of abdominal obesity among females and 52.81% among males [33].

The TMT was used to assess cognitive function (processing speed and executive function). TMT has been reported to be influenced by age and education but not sex [35]. The mean score for TMT-A and TMT-B were 51.02 ± 34.28 and 99.86 ± 69.23 seconds respectively, with males performing better than females in both tests (*p* < 0.005). We found a significant association between BMI and TMT-A (processing speed) but not TMT-B (executive function). This is not surprising as increasing BMI has been found to be associated with poor cognitive function among obese children, adolescents and adults [6, 36]. Similarly, central obesity, was significantly associated with poor performance on both TMT-A and TMT-B (*p* < 0.05), consistent with findings from prior reports [8]. This signifies that abdominal obesity may influence both the processing speed and executive function of an individual.

This study had several strengths, including the fact that it was conducted among a population with apparently no evident metabolic and cardiovascular abnormalities. It is however limited by the cross-sectional study design making it difficult to establish causal relationships between variables as well as extrapolating results to other populations. Nevertheless, it demonstrates that the association between obesity and cognitive function found in this study is more likely to be unbiased by comorbidities of obesity. The study may have been subject to response bias as participants were excluded (based on their self-report) from the study, if they had any neurological condition or physical disability that may affect assessment. Engaging a certified professional to ascertain the presence or otherwise of neurological conditions or physical disabilities would be beneficial for future studies. Further, BIA is influenced by factors such as hydration status, food intake and skin temperature. Although the assessment was done to mimic clinical assessments in Ghana as much as possible, the aforementioned factors were not taken into consideration in the data collection procedures and may be a source of bias. Future studies may consider assessing study participants under standardized conditions such as particular time of the day, a specified period after eating and drinking etc. Furthermore, though TMT is considered a valid and accurate assessment tool for executive function and processing speed, future studies should consider additional evaluation tools to explore broader aspects of cognitive function.

## Conclusion

The present study shows that adiposity may be related to certain aspects of cognitive function regardless of the weight or BMI. Increase in muscle mass may have a positive influence on cognitive function. Further more, there is a possible non-linear relationship between BMI, WHR and cognitive function (processing speed and executive function). Therefore more robust studies considering a larger sample size, inflammatory markers and multiple evaluation tools are needed to further clarify these associations. Policies and interventions targeting obesity must consider the cognitive or psychological influences of the condition. In addition, obesity interventions should focus on overall body composition, particularly adiposity, rather than weight alone.

## Data Availability

The datasets used and/or analysed during the current study are available from the corresponding author on reasonable request.

## Abbreviations

BMI: Body mass index
WHR: Waist to hip ratio
TMT: Trail making test
BIA: Bioelectric impedance analyser
DXA: Dual-energy x-ray absorptiometry
UG: University of ghana
UGEL: University of ghana enterprises limited
WHO: World health organization
BIA: Bioelectrical impedance analysis
SD: Standard deviation
SPSS: Statistical package for social sciences
